# Bovine MHC class II DR molecule plays a key role in bovine leukemia virus (BLV)-induced lymphoma

**DOI:** 10.1186/1742-4690-8-S1-A7

**Published:** 2011-06-06

**Authors:** Yoko Aida, Shin-nosuke Takeshima, Yuki Matsumoto, Taku Miyasaka, Yoshiyuki Miyazaki, Yoshihiro Tanabe, William C Davis, Kosuke Okada

**Affiliations:** 1 Viral Infectious Diseases Unit, RIKEN, 2-1 Hirosawa, Wako, Saitama, 351-0198, Japan; 2 Maebashi Institute of Animal science, Livestock Improvement Association of Japan; 3 Department of Veterinary Microbiology and Pathology, Washington State University, Pullman, WA, 99164-7040, USA; 4 Department of Veterinary Pathology, Faculty of Agriculture, Iwate University, Morioka, 020-8550, Japan

## 

BLV is the etiological agent of enzootic bovine leukosis (EBL), which emerges as B-cell lymphomas. Previously, we found one of tumor-associated antigens (TAAs), which is a useful tool not only for diagnosing EBL but also for screening of BLV-infected cattle with the potential to develop tumor in the future, by using the monoclonal antibody (MAb) c143. We here performed biochemical characterization of c143 TAA such as two-dimensional electrophoresis, sequential immunoprecipitation and tryptic peptide mapping with c143 MAb as well as MAbs against bovine major histocompatibility complex (BoLA) class II antigens, and flow cytometry and immunofluorescence staining of cells transiently transfection with a combination of BoLA-DRA, -DRB3, -DQA and -DQB cDNAs. First, it was found that the c143 TAA is BoLA-DR consisting of alpha and beta chains. Furthermore, the altered expressions of c143 TAA were observed in infected animal with lymphoma at various stages as follows: 1) The number of cells positive for the c143 TAA and 2) glycosylation of the beta chain increased with the progression of EBL. 3) The beta chain was specifically phosphorylated at serine residue(s) during the lymphoma stage. Finally, we tested an association of microsatellite markers using 86 cattle with lymphoma and 320 asymptomatic cattle. We identified that seven genomic markers which located in chromosomes 1, 3, 9, 15 , 21, 23 and 24, and, in addition, BoLA-DRB3 gene in chromosomes 23, showed suggestive linkage to development of the BLV-induced EBL. Collectively, our results strongly indicate that BoLA-DR plays a key role in BLV-induced diseases progression.

